# Cytome micronucleus assays with a metabolically competent human derived liver cell line (Huh6): A promising approach for routine testing of chemicals?

**DOI:** 10.1002/em.22254

**Published:** 2018-11-08

**Authors:** Miroslav Mišík, Armen Nersesyan, Claudia Bolognesi, Michael Kundi, Franziska Ferk, Siegfried Knasmueller

**Affiliations:** ^1^ Institute of Cancer Research, Department of Internal Medicine 1 Medical University of Vienna Vienna Austria; ^2^ Environmental Carcinogenesis Unit Ospedale Policlinico San Martino Genoa Italy; ^3^ Center for Public Health, Department of Environmental Health Medical University of Vienna Vienna Austria

**Keywords:** Huh6, micronuclei, nucleoplasmatic bridges, serum

## Abstract

One of the main problems of *in vitro* genotoxicity tests is the inadequate representation of drug metabolizing enzymes in most indicator cell lines which are currently used. We identified recently a human derived liver cell line (Huh6) which detected induction of DNA damage by representatives of different groups of promutagens without enzyme mix and showed that these cells are more suitable in terms of reproducibility and sensitivity as other currently used liver derived lines. We developed a protocol for micronucleus (MN) cytome assays with these cells and validated the procedure in experiments with representatives of different groups of directly and indirectly acting genotoxic carcinogens (MMS, cisplatin, PhIP, IQ, NDMA, B(a)P, AFB1, etoposide, and H_2_O_2_). The optimal cytochalasin B concentration in combination with 48 hr treatment was found to be 1.5 μg/mL and leads to a cytokinesis block proliferation index in the range between 1.7 and 2.0. The morphological characteristics of different nuclear anomalies which reflect DNA damage (MN, nuclear bridges, and buds) and their baseline frequencies in untreated cells were characterized, and the rates which are required to cause significant effects were calculated. All compounds caused dose dependent induction of MN when the cells were treated for 24 hr, longer and shorter exposure times were less effective. Experiments with different serum levels (fetal bovine serum [FBS]) showed that 10% FBS in the medium (instead of 4%) causes a substantial increase of the sensitivity of the cells. Our results indicate that the new protocol is a promising approach for routine testing of chemicals. Environ. Mol. Mutagen. 60: 134–144, 2019. © 2018 The Authors. *Environmental and Molecular Mutagenesis* published by Wiley Periodicals, Inc. on behalf of Environmental Mutagen Society.

## INTRODUCTION

One of the crucial problems of toxicity studies with mammalian and human cell lines is caused by inadequate representation of drug metabolizing enzymes which convert xenobiotics to DNA reactive metabolites and catalyze their detoxification. In the 1970s, an enzyme liver homogenate was developed by Malling (1971). This so called “S9 mix” contains active phase I enzymes (but no phase II enzymes) and is commonly used in routine screening tests with bacterial indicators and mammalian cell lines (Kirkland 1990). These models reflect the metabolism of chemicals in the human body only partly and may cause misleading results (Fowler et al., 2012). As a consequence, experiments with rodents are performed which could be possibly avoided to a certain extent by use of more reliable *in vitro* tests. One of the main limiting factors is the high number of false positive results (Fowler et al., 2014) which is probably a consequence of underrepresentation of detoxifying enzymes in the currently available indicator cells. A possible solution is the use of specific human derived liver cell lines which have retained the activities of a variety of drug metabolizing phase I and II enzymes (Knasmuller et al., 2004; Winter et al., 2008; Le Hegarat et al., 2010). We showed in a recent investigation in single cell gel electrophoresis (SCGE) experiments (Waldherr et al., 2018) that the human liver line Huh6, which was never used in genotoxicity studies before, detects representatives of a broad variety of DNA reactive genotoxins which require metabolic activation. Comparisons with results obtained with other liver lines which are used in genetic toxicology such as HepG2, HepaRG, Hep3B, and HCC1.2 showed that Huh6 cells are equally or more sensitive and/or that the experiments have a better reproducibility (Waldherr et al., 2018).

These promising results stimulated us to develop a standardized protocol for micronucleus (MN) cytome assays with these cells and to evaluate its suitability for the detection of different groups of genotoxic carcinogens which are either directly active or require activation *via* different metabolic pathways. The MN cytome assay is one of the most widely tests in genetic toxicology (Kirsch‐Volders et al., 2011; Fenech et al., 2013) and an OECD guideline for MN assays with mammalian cells in routine testing of chemicals has been developed (OECD 2014).

In the first series of experiments, we studied the growth kinetics of the cells. Subsequently, the ideal treatment period and the optimal cytochalasin B (Cyt B) concentration were determined. In further experiments, we established the background rates of different nuclear anomalies (MN, nuclear buds – NBuds and nuclear bridges – NBs) and determined the cytokinesis block proliferation index (CBPI) in untreated cultures. Next, a picture gallery showing the morphological characteristics of the cells and of the different nuclear anomalies was established and several series of experiments with representatives of different groups of model mutagens were conducted (see Table 1). In order to define the optimal treatment periods, different exposure times were tested. It is known, from experiments with other liver cell lines that exposure periods may increase the sensitivity of liver derived cells (Natarajan and Darroudi 1991), possibly as a consequence of induction of activating enzymes. The last experimental series concerned the investigation of the impact of different serum concentrations on the sensitivity of the cells.

**Table 1:** Use, Occurrence and Mode of Action of the Different Model Compounds which were Tested in the Present Study

Test compound (abbreviation)	Occurrence/use	Mode of action/IARC classification	References
**Directly acting**		
Cisplatin (CDDP)	Cytostatic drug	Crosslinking agent; causes different types of DNA damage (gene mutations and CA); IARC: Group 2A	Jackson et al. (1996)
Etoposide (Etop)	Cytostatic drug	Topoisomerase inhibitor; causes DNA damage (gene mutations and CA); IARC: Group 1	Jackson et al. (1996)
Hydrogen peroxide (H_2_O_2_)	Bleaching, industrial processes – causes oxidation	Decomposition leads to formation OH^**˙**^ and O_2_^**˙**^ radicals; causes oxidation of DNA; IARC: Group 3	Menghini (1988)
Methyl methanesulfonate (MMS)	Cytostatic drug	Methylation of DNA bases; causes gene mutations and CA; IARC: Group 2A	IARC (1999)
**Indirectly acting mutagens (promutagens)**		
Aflatoxin B1 (AFB1)	Mycotoxin produced by *Aspergillus flavus* and *Aspergillus parasiticus*; occurs in peanuts, maize, *etc*.	Formation of epoxide causing guanine adducts; activation: CYP1A2, 2B6, 3A4, 3A5, 3A7; detoxification: GSTs; IARC: Group 1	IARC (1993)
Benzo(a)pyrene (B(a)P)	Polycyclic aromatic hydrocarbon – combustion product of organic material (eg, tobacco smoke, ambient air, grilled/broiled and smoke‐cured meats)	Formation of epoxide which causes guanine adducts; activation: CYP1A1, 1A2, and 1B1; detoxification: GSTs, UGTs and SULTs; IARC: Group 1	IARC (2010)
*N*‐nitrosodimethyl – amine (NDMA)	Formed in stomach (*in vivo*) and in tobacco smoke, meat and fish products	Methylation of the DNA; activation: CYP2E1; IARC: Group 2A	IARC (1978)
2‐Amino‐3‐methyl‐3H‐imidazo[4,5‐f]quinoline (IQ)	Heterocyclic amine found in grilled/boiled meat and fish	Formation of guanine adducts, formation of DNA‐reactive metabolites which cause DNA‐adducts; activation: CYP1A2 and NAT; detoxification: GSTs and SULTs; IARC: Group 2A	IARC (1993)
2‐Amino‐1‐methyl‐6‐phenylimidazo[4,5‐b]pyridine (PhiP)	Heterocyclic amine ‐ cooked beef, pork, chicken and fish products	Formation of guanine adducts; activation: CYP1A2 and SULT; detoxification: GSTs; IARC: Group 2B	IARC (1993)

CYP – cytochromes; CA – chromosomal aberrations; GSTs – glutathione S‐transferases; SULTs – sulfotransferases; UGT – glucuronosyltransferase; IARC classification according to IARC (2018).

On the basis of the results, a standardized protocol was developed which can be used in future experiments.

## MATERIALS AND METHODS

### Chemicals

Aflatoxin B1 (AFB1, CAS‐No. 1162 65 8), benzo(a)pyrene (B(a)P, CAS‐No. 50‐32‐8), hydrogen peroxide (H_2_O_2_, CAS‐No. 7722‐84‐1), *N*‐nitrosodimethylamine (NDMA, CAS‐No. 62–75‐9), methylmethane sulfonate (MMS, CAS‐No. 66‐27‐3), cisplatin (CDDP, CAS‐No. 15663‐27‐1), and cytochalasin B (Cyt B) were purchased from Sigma‐Aldrich (St Louis, MO). 2‐Amino‐3‐methyl‐3H‐imidazo[4,5‐f]quinoline (IQ, CAS‐No. 76180‐96‐6) came from Toronto Research Chemicals (Toronto, ON) and 2‐amino‐1‐methyl‐6‐phenylimidazo[4,5‐b]pyridine (PhIP, CAS‐No. 105650‐23‐5), etoposide (Etop, CAS‐No. 33419‐42‐0) from Santa Cruz Biotechnology, Inc. (Dallas, TX). Inorganic salts, dimethyl sulfoxide (DMSO), fetal bovine serum (FBS), Dulbecco's Phosphate Buffered Saline (DPBS), Roswell Park Memorial Institute Medium 1640 (RPMI) were purchased from Sigma‐Aldrich (Steinheim, Germany). DiffQuick was ordered from Labor+Technik, (Salzburg, Austria), trypsin‐EDTA from Life Technologies (Karlsruhe, Germany).

### Cell Origin and Cultivation

Huh6 cells were obtained from W. Mikulits (Medical University of Vienna, Austria). The identity of the cell line was verified by short tandem repeat analyses (van Zijl et al., 2011). The cells were cultivated at 37°C, 96.0% humidity in 5.0% CO_2_ in TC‐treated flasks (Sigma‐Aldrich, in RPMI medium (with 4% of 10% FBS) as described in a previous publication (for details see Waldherr et al., 2018) and were routinely checked for mycoplasma contaminations by PCR (Mycoplasma Plus PCR Primer Set, Cat. No. 302008, Agilent Technologies, Santa Clara, CA). The media were changed every 4–5 days, when the cultures had reached confluence, subsequently the cells were washed with DPBS, detached with trypsin/EDTA, centrifuged and sub‐cultured.

### Determination of the Growth Kinetics

The proliferation of the cells was studied by use of a CASY® Cell Counter and Analyzer System (TTC‐2EA‐1087, Schärfe System GmbH, Reutlingen, Germany) which was used as described by the manufacturer (Schärfe‐System GmbH). Cell lines were grown in Petri dishes (Ø 6 cm, Sigma‐Aldrich) in 5.0 mL medium; *ca*. 5 × 10^5^ cells were seeded in each dish at the start of the experiments. After 24 hr intervals, the cells were detached with trypsin–EDTA (Szabo‐Scandic, Vienna, Austria) and 50 μL of the suspensions were transferred to CASY‐cups (OLS OMNI Life Science GmbH & Co. KG, Bremen, Germany). For each experimental point, three plates were evaluated. On the basis of the results, means and standard deviations (SDs) were calculated. Nonlinear fits with exponential growth equations (least square fits) were calculated to determine the doubling time.

### Cytochalasin B Treatment

The cells were seeded (3.5–4.0 × 10^5^/well) in 6‐well plates (3.0 mL of 4% FBS RPMI) for 12 hr. Different time periods (24–72 hr) were tested in combination with 3.0 μg/mL Cyt B. Subsequently, the 48 hr period was used to test different doses concentrations of Cyt B (1.5–6.0 μg/mL).

The formation of binucleated cells was monitored in experiments in which the cells were exposed to 3.0 μg/mL Cyt B for different time period. Subsequently, we determined the division indices after treatment with different concentrations of Cyt B (1.5–6.0 μg/mL) for 48 hr. We tested different concentrations since it can be not excluded that the drug may cause adverse effects; therefore, we tried to keep its concentration as low as possible.

The CBPIs were calculated with 500 cells according to OECD guideline #487 (2014) with the formula CBPI = [M1 + 2M2 + 3(M3 + M4)]/*N* (*N* is the total number of scored cells, M1–M4 refer to the number of cells with one to four nuclei). All experiments were performed in duplicate.

Untreated and treated cells were fixed and stained with DiffQuick according to the instructions of manufacturers. Subsequently, photographic images of selected anomalies were made with a light microscope (Leica DM RXA) at 400× magnification using a photocamera DFC450C (Leica, Milan, Italy).

### Experiments with Selected Mutagens and Determination of the Background Frequencies of Different Nuclear Anomalies

The model compounds were dissolved either in DMSO (AFB1, B(a)P, IQ, PhiP, and Etop) or in medium (NDMA, MMS, and CDDP). 3.8–4.0 × 10^5^ cells per well were seeded into 6‐well plates with 3 mL of RPMI (4% FBS) for 12 hr, then dilutions of stock solutions of the diagnostic mutagens were added for different time periods (4, 24, and 48 hr). In each experimental series solvent controls were included. Subsequently, the cells were washed twice with 3.0 mL medium. Subsequently, new medium with Cyt B (1.5 μg/mL) was added for 48 hr. Finally, the cells were washed and trypsinised. Afterwards the cells were washed twice with 8.0 mL RPMI and collected on slides by cytocentrifugation (600 rpm, 5 min). The slides were air dried, fixed, and stained with DiffQuick according to the instructions of the manufacturer. For each experimental point, two cultures were tested per dose. From each culture ≥1,000 BN cells were evaluated under 400‐fold magnification (Nikon Photophot‐FXA, Tokyo, Japan). At least 2,000 binucleated cells were analyzed per dose for the determination of the different nuclear anomalies (MN, NBuds, and NBs).

The dose ranges for most chemicals (AFB1, B(a)P, IQ, PhiP, NDMA, H_2_O_2_, and MMS) were chosen on the basis of results obtained with Huh6 cells in comet assays (Waldherr et al., 2018); for other agents (CDDP and ET) they were selected on the basis of earlier MN studies with HepG2 (Pezdirc et al., 2013).

### Investigation of the Impact of Serum on the Sensitivity of the Cells

The experiments were performed as described in the previous chapter. The cells were exposed only to one dose of the different mutagens and were cultivated after the seeding in 6‐well plates which contained 4% or 10% FBS in RPMI, respectively.

### Statistical Analyses

Results are reported as means ± standard deviations (SD) from two cultures and from four cultures for controls. Data were evaluated by generalized linear model with Poisson counts. Chi‐square tests for overdispersion were applied. Results for the different doses were compared against controls by linear contrasts with Bonferroni–Holm correction if necessary. Results obtained with 10% and 4% serum were compared also by linear contrasts, however, after subtraction of the respective control results. *P* values ≤0.05 were considered statistically significant.

## RESULTS

**Figure 1A,B.**
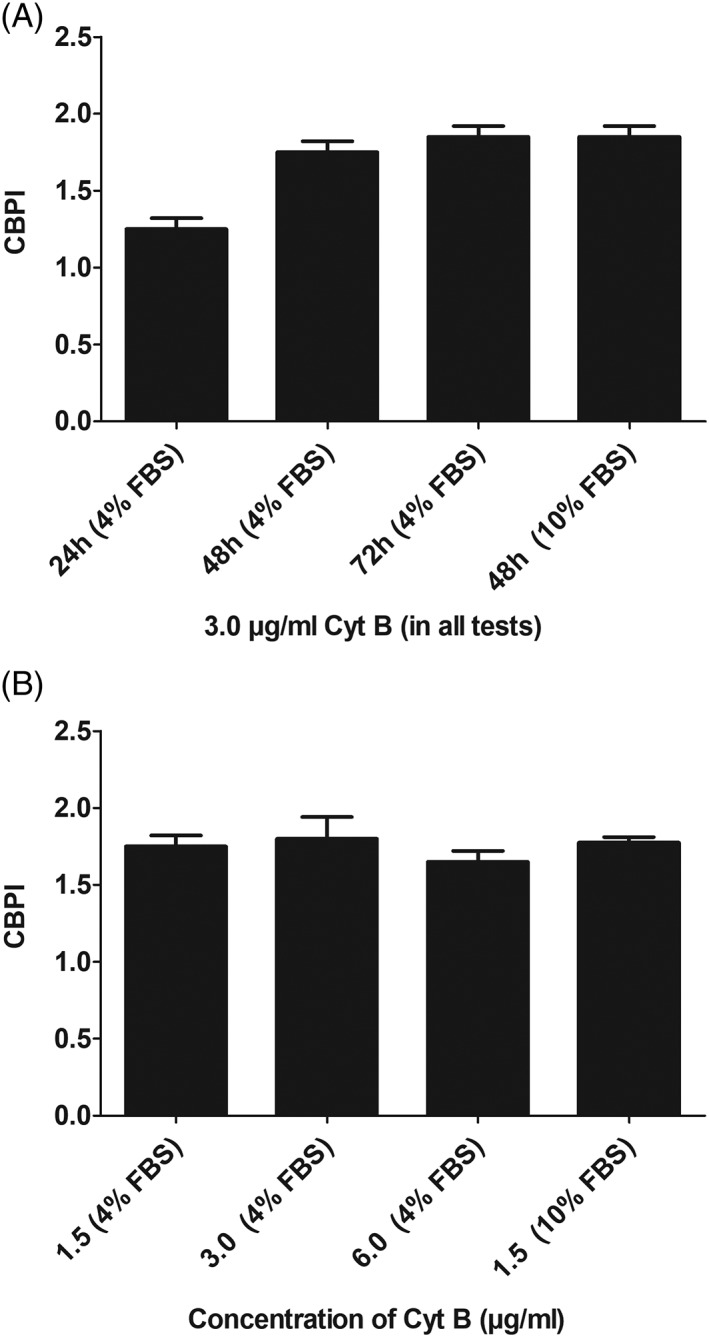
Impact of Cyt B treatment on the proliferation of Huh6 cells. A: Impact of different treatment periods in combination with 3.0 μg/mL Cyt B on CBPI values. B: Impact of different Cyt B concentrations on the CBPI values (exposure time 48 hr). Data show means ± SD of results obtained with two cultures per experimental point. From each culture at least 500 cells were evaluated.

In order to evaluate the MN frequencies in binoculated cells (BNC), it is essential to add Cyt B for a sufficiently long time period. According to OECD guideline #487 (2014), Cyt B should be added for at least 1.5–2 cell cycles, furthermore, it is stated in the protocol for experiments with lymphocytes that the NDI values should be in the range between 1.3 and 2.2 (Fenech 2007). The growth curve for Huh6 cells is shown in Supporting Information Figure S1; on the basis of these measurements, the average doubling time of the untreated cells was calculated (ca. 35 hr). Figure 1A,B. shows the CBPIs which were found after addition of Cyt B (3.0 μg/mL) for different time periods (24–72 hr). It can be seen that the values were in the range between 1.3 and 1.9. These results are based on the cultivation of the cells in 4% serum; a higher serum concentration (10%) had no impact on the CBPI values (Fig. 1A,B.). In further experiments we tested the effect of different Cyt B concentrations on the growth kinetics. It can be seen in Figure 1A,B. that a significant decrease was observed with the highest concentration (6.0 μg/mL). Again the increase of the serum concentration (from 4% to 10% FBS) had no impact on the division rate of the cells.

### Morphological Characteristics of Huh6 Cells and Specific Nuclear Anomalies

**Figure 2:**
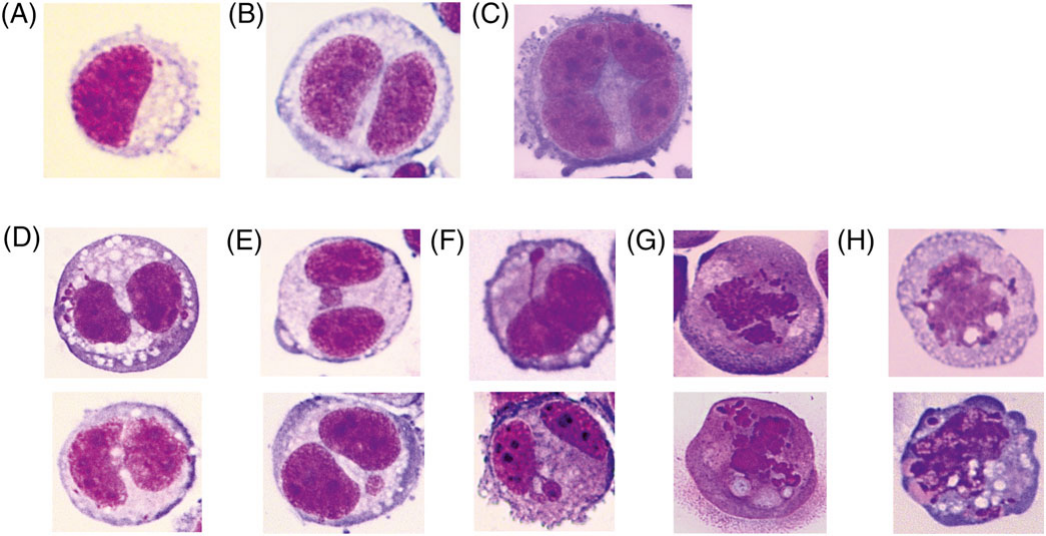
Photographic images of the cells and different nuclear anomalies (NBs – nucleoplasmic briges; NBuds – nucleoplasmic buds), stain Diff Quick, magnification 400×, (A) mononucleated epithelioid cell with some cytoplasmic vacuoles and few desmosomes; (B) binucleated epithelioid cell; (C) multinucleated epithelioid cell with desmosomes; (D) binucleated epithelioid cells with some cytoplasmic vacuoles and one or two nucleoplasmic bridges; (E) binucleated epithelioid cells with a micronucleus in different position; (F) binucleated epithelioid cells with nuclear buds; (G) mononucleated apoptotic cells; (H) mononucleated necrotic cells.

Figure 2 shows photographic images of the morphology of Huh6 cells and of other nuclear anomalies. The cells have epithelial features and are characterized by desmosomes and glycogen granules in the cytoplasm. Nuclear anomalies which are indicative for genotoxic effects (MN, NBs, and NBuds) are shown in Figure 2; their morphology is similar to that observed in human lymphocytes and the scoring criteria which were developed for these cells can be used (for details see Fenech 2007).

### Background Frequencies of Different Nuclear Anomalies

Table 2 shows the background rates of the different nuclear anomalies. The cells were cultivated either in 4% or in 10% serum and the frequencies were determined after treatment with 1.5 μg/mL Cyt B for 48 hr.

**Table 2:** Number of Anomalies per 1,000 Binucleated Cells and 95% Confidence Intervals Estimated by Poisson Regression^a^

Endpoint	Condition	*n*/1,000	95% CI		SE	*P* value	4 hr	24 hr	24 hr (10%)	48 hr
MNi^b^	4 hr	16.00	13.45	19.03	1.41	0.678	‐	0.482	0.711	0.567
	24 hr	14.63	12.20	17.53	1.35		0.482	‐	0.283	0.896
	24 hr (10%)	16.75	14.14	19.84	1.45		0.711	0.283	‐	0.346
	48 hr	14.88	12.43	17.80	1.36		0.567	0.896	0.346	‐
Total MNi^c^	4 hr	16.75	14.14	19.84	1.45	0.549	‐	0.806	0.373	0.665
	24 hr	16.25	13.68	19.30	1.43		0.806	‐	0.255	0.852
	24 hr (10%)	18.63	15.86	21.87	1.53		0.373	0.255	‐	0.185
	48 hr	15.88	13.34	18.89	1.41		0.665	0.852	0.185	‐
NBuds	4 hr	13.25	10.95	16.03	1.29	0.001^d^	‐	0.575	0.002^d^	0.081
	24 hr	12.25	10.05	14.93	1.24		0.575	‐	<0.001^d^	0.021^d^
	24 hr (10%)	19.50	16.67	22.81	1.56		0.002^d^	<0.001^d^	‐	0.176
	48 hr	16.63	14.03	19.70	1.44		0.081	0.021^d^	0.176	‐
NB	4 hr	0.25	0.06	1.00	0.18	0.938	‐	1.000	1.000	0.564
	24 hr	0.25	0.06	1.00	0.18		1.000	‐	1.000	0.564
	24 hr (10%)	0.25	0.06	1.00	0.18		1.000	1.000	‐	0.564
	48 hr	0.13	0.02	0.89	0.13		0.564	0.564	0.564	‐

a Standard error from 4 experiments with duplicate counts.

b MNi – number of micronucleated cells in 1,000 binucleated cells.

c Total number of micronuclei in 1,000 binucleated cells. *P* value for comparison of all conditions and from individual comparisons.

d Data were evaluated by generalized linear model with Poisson counts. Chi‐square tests for overdispersion were applied. Stars indicate statistical significance (*P* ≤ 0.05).

For MN (either total numbers or MNated cells) and NBs no relevant differences were found across conditions. Somewhat higher values were observed at 24 hr and with 10% serum. This condition (24 hr and 10% serum) showed significantly higher counts for nuclear buds. Nuclear bridges were rarely detected and no difference was found between conditions.

Based on the background rates, the fold‐increase and the slopes that can be detected were computed. On the basis of the standard OECD (2014) guideline for *in vitro* MN experiments (three doses and a solvent control, with two cultures per experimental point), a 5% level of significance and no increase above background level for the lowest dose the following slopes (increases per dose increment, that is, per one dose factor or one dose addition for geometrically or arithmetically spaced doses, respectively) will have an 80% power to be detected by Poisson regression: for MNi 1.39, total MNi 1.37, NBuds 1.36, and NBs 3.64. These values reduce to 1.28, 1.26, 1.25, and 3.39, if already the first dose has a 10% increase above background levels. The fold‐increase that can be detected under identical conditions with a single dose is 1.54 for MNi, 1.51 for total MNi, 1.50 for NBuds, and 5.38 for NBs.

### Detection of Directly and Indirectly Acting Chemicals

The results which were obtained with a panel of indirectly and directly acting model compounds after different treatment periods are listed in Supporting Information Table 1A–I. With all nine chemicals, significant dose dependent induction of MN was found. Furthermore, we observed also in all experimental series induction of NBuds (at least with one exposure period), NBs were not induced.

**Figure 3:**
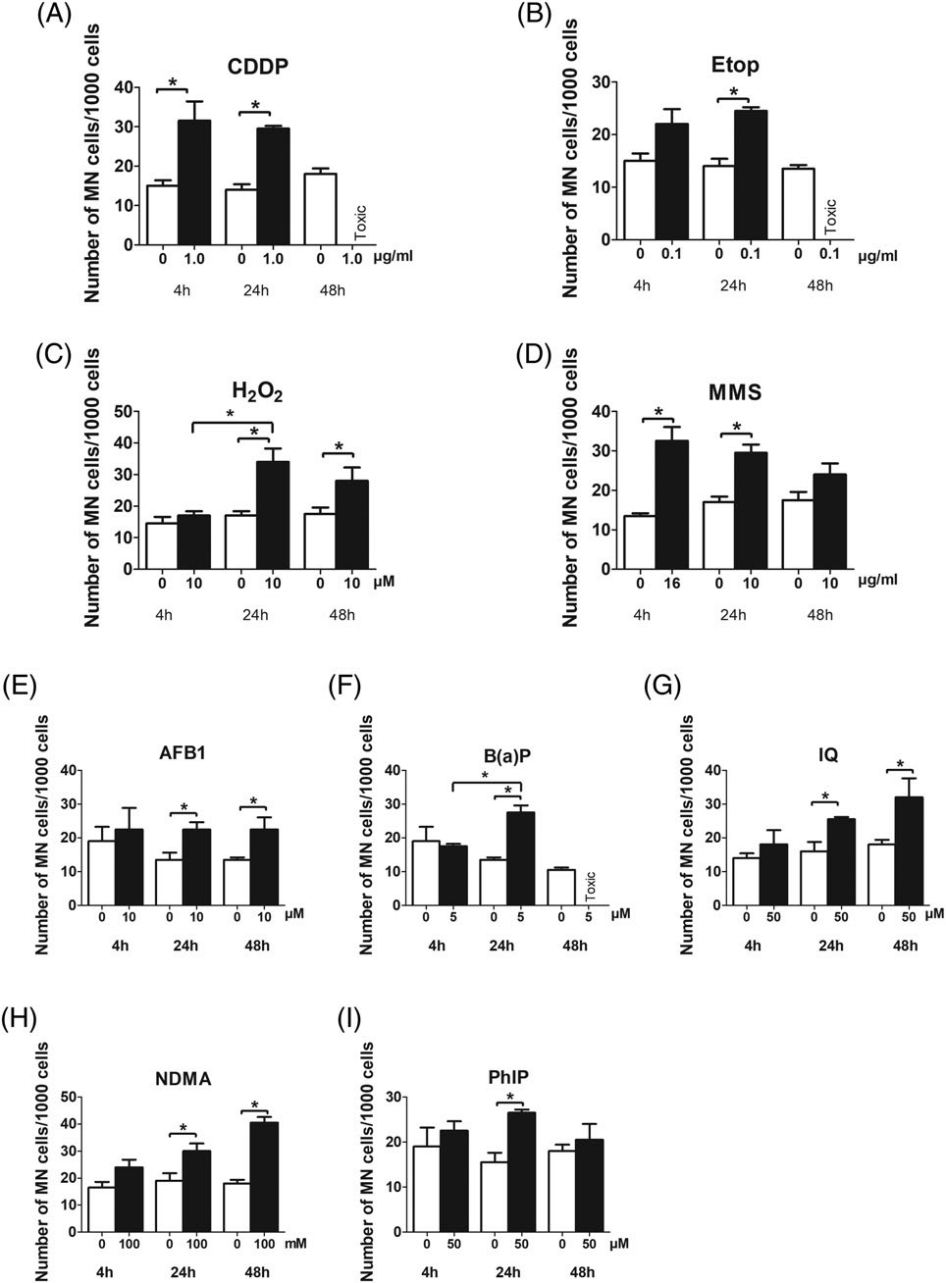
Induction of MN after treatment of Huh6 cells with different direct (A–D) and indirect (E–I) acting mutagens for different time periods. CDDP, H_2_O_2_, MMS, and NDMA were dissolved in medium, Etop, AFB1, B(a)P, IQ, and PhiP were dissolved in DMSO. Bars show means ± SD of results obtained in a representative experiment. Two cultures were treated per experimental point and from each, at least 1,000 BNC cells were evaluated. Data were evaluated by generalized linear model with Poisson counts. Chi‐square tests for overdispersion were applied. Stars indicate statistical significance (*P* ≤ 0.05).

The impact of different treatment periods on the MN frequencies is shown graphically in Figure 3. With indirectly acting promutagens (which require activation by phase I enzymes, Fig. 3E–I) we found optimal induction of MN after treatment for 24 and 48 hr. With directly acting compounds no differences were seen when the cells were exposed for 4 or 24 hr (Fig. 3A–D), except with H_2_O_2_, which did not cause an effect after 4 hr. After 48 hr we observed a slight decrease of the MN rates in H_2_O_2_ and MMS treated cultures in experiments with cytostatic drugs (Etop and CDDP) the MN frequencies could be not evaluated as a consequence of low CBPI values.

### Impact of Different Serum Concentrations on the Results of MN Experiments

**Figure 4:**
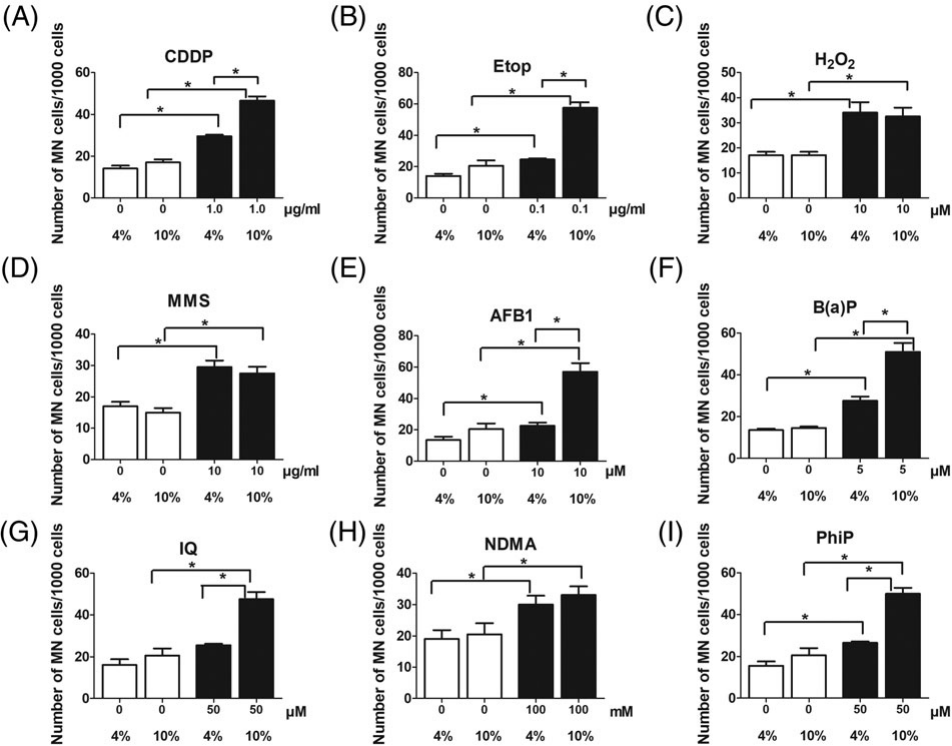
Impact of different serum conditions (4% and 10%) on MN induction by various model mutagens. The cells were tested with the chemicals for 24 hr and Cyt B (1.5 μg/mL) was added for 48 hr. Bars show means ± SD of results obtained in a representative experiment; two cultures were treated per experimental point and from each at least 1,000 BNC cells were evaluated. Data were evaluated by generalized linear model with Poisson counts. Chi‐square tests for overdispersion were applied. Stars indicate statistical significance (*P* ≤ 0.05).

The results which were obtained in subsequent MN experiments with 10% serum and 24 hr treatment are summarized in Figure 4. Again positive results were obtained with all model compounds; interestingly we found with certain mutagens a dramatic increase of the MN rates. As shown in Figure 4A–I the MN frequencies were not increased in control cultures and in experiments with directly acting chemicals such as MMS and H_2_O_2_. However, a pronounced increase of the MN frequencies was observed with Etop and CDDP after cultivation in 10% serum. Also with the promutagens (AFB1, B(a)P, IQ, and PhiP) except with NDMA, stronger effects were found with the higher serum concentration. The CBPI values are listed in detail in Supporting Information Tables 1A–I and 2. It can be seen that they were in all experimental series in the acceptable range that is, between 2.0 and 1.4 and MN were evaluated only when the toxicity was below 60%.

## DISCUSSION

This study describes the development of a standardized protocol for MN assays with the human derived liver cell Huh6. We defined the optimal concentration for and treatment time with Cyt B and studied morphological characteristics and background frequencies of different nuclear anomalies. Furthermore, we investigated the sensitivity of the cells toward different model mutagens and determined the most appropriate exposure period and the ideal serum concentration.

The optimal treatment period with Cyt B was in the present study 48 hr. The CBPIs which were found under this condition were in the range between 1.7 and 2.0. These values are in the range suggested by Fenech (1.3–2.2) for experiments with different mammalian cell lines (Fenech 2007). An increase of the Cyt B concentrations from 1.5 to 6.0 μg/mL had no impact on the division rates of cells (see Fig. 1A,B.).

The background values of different nuclear anomalies and the respective CBPI (NDI) values which were found in different human derived liver cell lines are compared in Table 3. It can be seen that the MN frequencies are similar in Huh6 and HepG2, NBs, and NBuds are higher in latter cell line. Furthermore, it is notable that the frequencies of NBuds are higher in Huh6 as in lymphocytes. A possible explanation may be that Huh6 are transformed cancer cells, while lymphocytes are primary cells.

**Table 3:** Examples of Background Frequencies of Nuclear Anomalies in Different Human Derived Cells

Endpoint	BN‐MN	MN	NBs	NBuds	CBPI	References
HepaRG	31.0	n.s.	n.s.	n.s.	1.9	Le Hegarat et al. (2010)
HepaRG	31.0	n.s.	n.s.	n.s.	n.s.	Le Hegarat et al. (2014)
HepG2	14.5	13.8	2.3	39.0	1.8	Pezdirc et al. (2013)
HepG2	n.s.	21.0	n.s.	n.s.	n.s.	Knasmuller et al. (1999)
Hep3B	n.s.	26.6	n.s.	n.s.	n.s.	Majer et al. (2004)
Human lymphocytes	n.s.	16.9	2.8	6.1	1.7	Lee et al. (2015)
Human lymphocytes	n.s.	5.1	1.2	3.6	2.0	Gajski et al. (2018)
Human lymphocytes	7.0	7.0	3.0	0.0	n.s.	Garaj‐Vrhovac et al. (2008)
Human lymphocytes	4.3	5.0	0.5	0.0	1.6	Cayir et al. (2014)
TR146	1.9	1.9	1.6	1.7	2.1	Al‐Serori et al. (2017)
Huh6	15.5	17.3	0.3	13.8	1.9	Present study

n.s. – not specified; data are means of three independent experiments; BN‐MN/1,000 – number of micronucleated cells in 1,000 binucleated cells. MN/1,000 – total number of micronuclei in 1,000 binucleated cells; NBs/1,000 – number of nucleoplasmatic bridges in 1,000 binucleated cells; NBuds/1,000 – number of nucleoplasmatic buds in 1,000 binucleated cells; CBPI – cytokinesis block proliferation index.

Table 3 summarizes examples for background frequencies obtained with different human cell types., With TR146, HepaRG and Hep3B only few MN studies were realized whereas a larger database is available for human lymphocytes and HepG2 (Knasmuller et al., 2004; Fenech 2007; Pezdirc et al., 2013). We found no significant differences of the background rates of MN in independent experimental series (see Table 2). On the basis of these results it was possible to calculate the numbers of the different nuclear anomalies (MN, NBuds, and NBs) which are needed to cause a significant effects in experiments with Huh6 cells, when the tests are conducted according to OECD guideline #487 (2014).

The morphological characteristics of Huh6 cells are shown in Figure 2. In agreement with Doi (1976) who isolated this cell line in 1976 from a hepatoblastoma of a 1‐year‐old boy, we found that the cells have epithelioid characteristics and contain many cytoplasmatic granules and some vacuoles and desmosomes. The size and shape of the cells is quite homogeneous. The morphology of nuclear anomalies (MN, NBs, and NBuds) and also apoptotic and necrotic cells is similar to that found in lymphocytes and the criteria which were developed for these cells also can be applied for scoring Huh6 cells.

A very important parameter which has an impact on the reproducibility of MN experiments with mammalian cell lines is the chromosomal stability of the indicator cells (Kirkland et al., 2007). For example it was shown that fluctuations of the chromosome numbers in CHO cells have a pronounced effect on their sensitivity toward clastogens (Kirkland et al., 2007). In this regard it is notable that we monitored the chromosomal number in Huh6 cells (Waldherr et al., 2018) 11 months before the realization of the present study and again after 10 passages before we started MN experiments (first analyses 82–86 sec analyses 82–87 chromosomes). However, further monitoring of the stability of the cells will be conducted in future. It is notable that the cells have an intact inducible *p53* (Waldherr et al., 2018). This characteristic is relevant for their use in genotoxicity testing as it was shown that it has an impact on the reliability of the indicator cell lines (Kirkland et al., 2007; Pfuhler et al., 2011). We monitored the activities of a variety of CYPs and phase II enzymes and found clear evidence that enzymes which are involved in the activation/detoxification of genotoxic carcinogens (e.g., EROD, MROD, BROD, PROD, as well as GST, GPx, and NAT) are active in Huh6 cells (Mišík M, Nersesyan A, Ropek N, Huber W, Haslinger E, Knasmueller S. 2018. Use of human derived liver cells for the detection of genotoxins in comet assays. Mutat Res. submitted). However, comparisons with earlier findings indicate that their levels are higher in primary hepatocytes.

As described in Supporting Information Table 1 we obtained with all diagnostic mutagens positive results and clear dose response effects were observed. For reasons of comparison we summarized the LOECs which were obtained in MN experiments with various human derived cell types in Table 4.

**Table 4:** Examples of Lowest Effective Concentrations (LOEC) of Various Model Compounds Found in Micronucleus Experiments with Different Human Derived Cell Types

Substance/	Cell type				
	Huh6	HepaRG	HepG2	Hep3B	Human lymphocytes
AFB1 (μM)	10.0	0.25 (Le Hegarat et al., 2010)	0.5 (Josse et al., 2012)	0.5 (Majer et al., 2004)	0.1 + S9 (Miller et al., 1998)
B(a)P (μM)	5.0	5.0 (Le Hegarat et al., 2010)	25.0 (Josse et al., 2012) 0.5 (Valentin‐Severin et al., 2003)	25.0 (Majer et al., 2004)	119.0 + S9 (Miller et al., 1998) 51.0 + S9 (Fowler et al., 2014)
PhiP (μM)	50.0	Negative (NOEC 320.0) (Le Hegarat et al., 2010)	25.0 (Majer et al., 2004)	Negative (NOEC 300.0) (Majer et al., 2004)	2.5 + S9 (Katic et al., 2010)
IQ (μM)	50.0	Negative (NOEC 500) (Le Hegarat et al., 2010)	300.0 (Knasmuller et al., 1999)	Not tested	2200.0 + S9 (Fowler et al., 2014) 250.0 + S9 (Katic et al., 2010)
MMS (μg/mL)	10.0	9.9 (Josse et al., 2012)	11.0 (Valentin‐Severin et al., 2003)	Not tested	51.0 (Fowler et al., 2014) 15.4 (Andreoli et al., 1999)
NDMA (μM)	1 × 10^5^	Not tested	3 × 10^4^ (Majer et al., 2004) 1 × 10^4^ (Valentin‐Severin et al., 2003)	Negative (NOEC 1.8 × 10^5^) (Majer et al., 2004)	Negative (NOEC 6.75 × 10^4^ + S9; Katic et al., 2010)
H_2_O_2_ (μM)	10.0	Not tested	Not tested	Not tested	100 (Andreoli et al., 1999)
Etop (μg/mL)	0.1	2.5 (Le Hegarat et al., 2014)	0.1 (Gajski et al., 2016)	Not tested	0.5 (Fowler et al., 2014)
CDDP (μg/mL)	0.5	Not tested	0.1 (Gajski et al., 2016)	Not tested	1.0 (Fowler et al., 2014)

S9 – metabolic activation mix; NOEC – highest no observable effect concentration tested.

It is evident that HepG2 cells which were frequently used in genotoxicity experiments are equally or more sensitive as Huh6. The main problem of experiments with HepG2 cells is the poor reproducibility; for example, the responses which were obtained in comet assays with different model compounds vary over a broad range (Waldherr et al., 2018) and the concentration which were required to obtain positive results with B(a)P in different labs in MN experiments vary over a broad range (Knasmuller et al., 2004). HepaRG are clearly more sensitive toward AFB1 but do not detect IQ and PhiP; furthermore, they are less sensitive to Etop and comparison of results obtained in SCGE experiments with H_2_O_2_ indicate that they are insensitive toward ROS induced DNA damage (Waldherr et al., 2018).

In order to define the optimal treatment time for MN experiments, we evaluated the effects of the model compounds after different exposure periods. This issue is of importance as DNA damage caused by direct acting compounds may be repaired; on the other hand it is known that indirect acting mutagens induce activating as well as detoxifying enzymes (Elovaara et al., 2007; Pezdirc et al., 2013). This effect may increase as a function of the treatment time. Figure 4A–D shows that the effects of most direct acting compounds (CDDP, Etop, and MMS) and also of AFB1 are similar regardless if the cells are treated for 4 or 24 hr, while more pronounced effects were observed with H_2_O_2_ and also with B(a)P, IQ, NDMA, and PhiP after 24 hr (or longer). Taken together, these findings indicate that exposure for 24 hr is ideal for MN experiments with Huh6 cells. Only few data from the literature are available which concern the impact of the treatment time of human cells in MN experiments. In studies with lymphocytes with S9 mix usually a short exposure time is used (3–6 hr) as the enzyme activities decline rapidly in the mix. Natarajan and Darroudi (1991) compared MN induction by different compounds in HepG2 cells after 1 and 28 hr. They found stronger effects with a longer exposure period with cyclophosphamide, NDMA and B(a)P, but clearly a less pronounced effect with MMS. In experiments with HepaRG (treatment for 1 or 7 days) the extent of MN formation was compound specific that is, stronger effects were detected with AFB1 and B(a)P with the longer exposure time, while no such effects were observed with MMS, dichlorodiphenyldichloroethylene, and noctazole (Josse et al., 2012).

We were surprised that no positive effects were obtained with H_2_O_2_ which causes direct oxidative DNA damage when we used the short treatment protocol (4 hr). A possible explanation may be that the overall cultivation period (H_2_O_2_ treatment + Cytochalasin B exposure) was in this case not sufficiently long (only 1.2 cell cycles) to cause MN formation as a consequence of oxidative DNA damage. The lack of a positive response seen with Etop after short term exposure may due to of the same reason. However, as shown in Figure 4, both compounds caused a significant increase in experiments with 4% serum (which is also significant when historical negative controls are considered) after 24 hr treatment. Furthermore, pronounced effects were obtained in experiments with 10% serum (see Fig. 4). The decrease of the MN numbers which were observed with MMS and H_2_O_2_ when the longest treatment interval (72 hr) was used may be a consequence of repair of the specific lesions caused by these agents (oxidative damage and alkylation of DNA bases).

Another relevant parameter which we studied is the effect of different serum concentrations. It was reported that the serum levels have an impact on the genotoxic and acute toxic properties of chemicals and nanoparticles by modulation of their bioavailability and/or alterations of the activity of drug metabolizing enzymes (Wiencke et al., 1984; Wang and Lee 2001; Gonzalez et al., 2014; Lubberstedt et al., 2015; Pisani et al., 2017). The serum effect was compound specific; it was observed with certain direct acting chemicals such as CDDP and Etop but not with other substances (MMS and H_2_O_2_). Clear effects were also detected with indirect compounds which are activated inside the indicator cells. Therefore, it is unlikely that direct protein binding effects in the incubation mix account for this phenomenon. It is possible that other factors, for example presence of growth hormones, in the serum lead to these effects. This important issue (which may be of general importance for genotoxicity tests with mammalian cells) should be investigated in more detail in further studies. Taken together findings show that the higher concentration is recommendable for routing testing.

Our results show that the protocol which we developed can be used to detect a variety of representative of genotoxic carcinogens and indicate that this approach may be useful for the routine testing of chemicals. We used in the present study potent well characterized direct and indirect acting mutagens which cause DNA damage *via* different mechanisms. In order to confirm the assumption that Huh6 cells are a useful tool for genetic toxicology testing, further validation is required. As mentioned above, misleading results (false positives) are often obtained in other *in vitro* assays. Therefore we will investigate the effects of such compounds and of other chemicals in MN experiments with Huh6 cells on the basis of the suggestions developed by experts of the ECVAM panel (Kirkland et al., 2016) in order to assess the specificity and sensitivity of MN‐studies with these cells.

## AUTHOR CONTRIBUTIONS

MM and AN conducted the experiments and evaluated the slides, CB provided microphotographs, MK carried out the statistical analyses, SK designed study and wrote the manuscript with MM. FF helped in the evaluation of the slides.

## CONFLICT OF INTEREST

The authors declared that they have no conflict of interest.

## Supporting information

Figure S1. Growth kinetics of Huh6 cells. Cells were seeded into Petri dishes (∅ 6 cm) and incubated under standard conditions (RPMI with 4% FBS, 37°C, 95% humidity, 5% CO_2_). The cell numbers were determined with a CASEY‐counter. Symbols indicate means ± S.D. obtained with three plates per experimental point.

Table S1. Impact of different exposure periods on the results of cytokinesis block MN assays with CDDP (4% FBS RPMI).^1^

Table S2. Results of cytome MN assays with different diagnostic mutagens (10% FBS RPMI)^1^
