# Supplementary material for: Cytome micronucleus assays with a metabolically competent human derived liver cell line (Huh6): A promising approach for routine testing of chemicals?
Source: Environ Mol Mutagen. 2018 Nov 8;60(2):134–44. doi: 10.1002/em.22254 (PMC6492180; doi:10.1002/em.22254)

S Figure 1

S Figure 1. Growth kinetics of Huh6 cells. Cells were seeded into Petri dishes (∅ 6 cm) and incubated under standard conditions (RPMI with 4% FBS, 37°C, 95% humidity, 5% CO_2_). The cell numbers were determined with a CASEY-counter. Symbols indicate means ± S.D. obtained with three plates per experimental point.


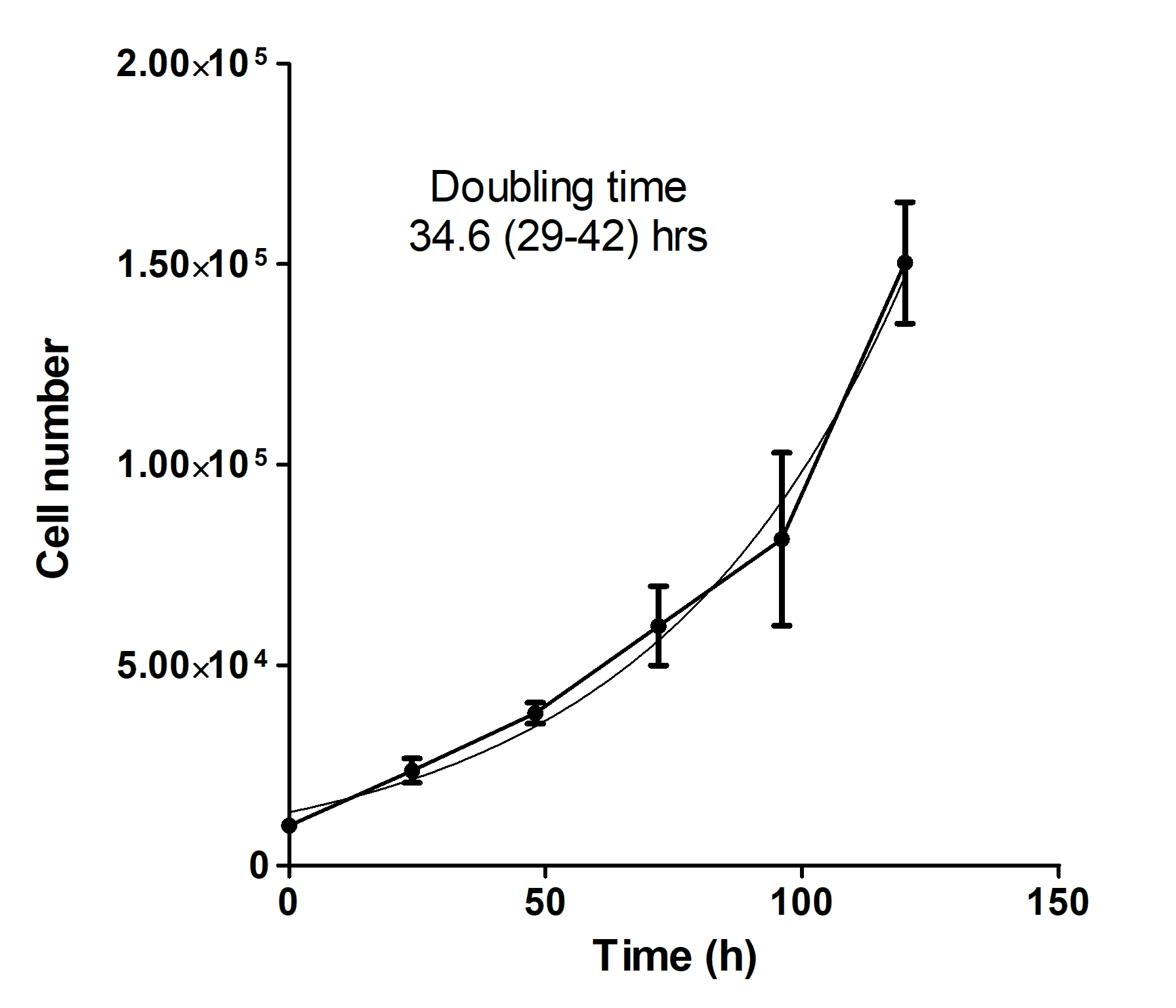

Supplement: Supplementary file 1 — Figure S1. Growth kinetics of Huh6 cells. Cells were seeded into Petri dishes (∅ 6 cm) and incubated under standard conditions (RPMI with 4% FBS, 37°C, 95% humidity, 5% CO2). The cell numbers were determined with a CASEY‐counter. Symbols indicate means ± S.D. obtained with three plates per experimental point. [file EM-60-134-s001.docx]
