# Supplementary material for: Cytome micronucleus assays with a metabolically competent human derived liver cell line (Huh6): A promising approach for routine testing of chemicals?
Source: Environ Mol Mutagen. 2018 Nov 8;60(2):134–44. doi: 10.1002/em.22254 (PMC6492180; doi:10.1002/em.22254)
Supplement: Supplementary file 2 — Table S1. Impact of different exposure periods on the results of cytokinesis block MN assays with CDDP (4% FBS RPMI).1 [file EM-60-134-s002.docx]

Table S1 A. Impact of different exposure periods on the results of cytokinesis block MN assays with CDDP (4% FBS RPMI).^1^

| **Time/µg/ml** | **Endpoint*** | | | | |
| --- | --- | --- | --- | --- | --- |
|  | **CBPI** | **BN-MN (‰)** | **MN(‰)** | **Buds (‰)** | **Bridges (‰)** |
| **4hrs** | | | | | |
| 0 | 1.9 ± 0.0 | 15.0 ± 1.4 | 15.0 ± 1.4 | 9.5 ± 2.1 | 0.0 ± 0.0 |
| 0.05 | 1.7 ± 0.1 | 16.0 ± 1.4 | 16.0 ± 1.4 | 15.5 ± 2.1 | 0.0 ± 0.0 |
| 0.1 | 1.6 ± 0.1 | 19.5 ± 0.7 | 20.0 ± 1.4 | 16.5 ± 2.1 | 0.0 ± 0.0 |
| 0.5 | 1.8 ± 0.1 | 19.5 ± 0.7 | 21.5 ± 0.7 | **19.0 ± 1.4*** | 0.0 ± 0.0 |
| 1 | 1.5 ± 0.1 | **31.5 ± 5.0*** | **33.5** ± **5.0*** | **34.0 ± 5.7*** | 3.5 ± 0.7 |
| **24hrs** | | | | | |
| 0 | 1.9 ± 0.0 | 14.0 ± 1.4 | 14.5 ± 2.1 | 15.5 ± 0.7 | 0.0 ± 0.0 |
| 0.1 | 1.8 ± 0.1 | 13.5 ± 0.7 | 14.0 ± 0.0 | 15.5 ± 0.7 | 0.0 ± 0.0 |
| 0.5 | 2.0 ± 0.0 | **25.5 ± 0.7*** | **34.0 ± 1.4*** | **30.5 ± 2.1*** | 2.5 ± 0.7 |
| 1 | 1.8 ± 0.0 | **29.5 ± 0.7*** | **35.0 ± 1.4*** | **36.0 ± 2.8*** | 5.0 ± 1.4 |
| **48hrs** | | | | | |
| 0 | 2.1 ± 0.1 | 18.0 ± 1.4 | 18.0 ± 1.4 | 23.5 ± 2.1 | 0.0 ± 0.0 |
| 0.1 | 1.6 ± 0.0 | 16.5 ± 0.7 | 17.0 ± 1.4 | 21.5 ± 2.1 | 0.0 ± 0.0 |
| 0.25 | 1.4 ± 0.1 | 20.0 ± 1.4 | 20.5 ± 0.7 | 20.5 ± 2.1 | 0.0 ± 0.0 |
| 1 | 1.2 ± 0.1 | **-** | - | - | - |

^1^50-60 000 cells were seeded in 6 well plates with 3 ml RPMI (4% FBS), subsequently different solutions of the test compound (dissolved in medium) were added. After treatment for different time periods, the cells was washed and processed as described in the Material and Method section. Numbers indicate means ± SD of values obtained in a representative experiment with two cultures per experimental point. From each culture 1000 binoculated cells were evaluated. BN-MN – number of micronucleated cells; MN **-** total number of micronuclei; CBPI – cytokinesis-block proliferation index

* indicate statistical significance (*p≤0.05).

Table S1 B. Impact of different exposure periods on the results of cytokinesis block MN assays with Etoposide (4% FBS RPMI).^1^

| **Time/**  **Dose (µg/ml)** | **Endpoint*** | | | | |
| --- | --- | --- | --- | --- | --- |
|  | **CBPI** | **BN-MN (‰)** | **MN(‰)** | **Buds (‰)** | **Bridges (‰)** |
| **4hrs** | | | | | |
| 0 | 1.8 ± 0.1 | 15.0 ± 1.4 | 15.0 ± 1.4 | 9.5 ± 2.1 | 0.0 ± 0.0 |
| 0.01 | 1.8 ± 0.1 | 14.5 ± 2.1 | 22.0 ± 2.8 | 12.0 ± 1.4 | 0.0 ± 0.0 |
| 0.05 | 1.8 ± 0.1 | 20.0 ± 2.8 | 20.0 ± 2.8 | 14.0± 5.7 | 0.0 ± 0.0 |
| 0.1 | 1.8 ± 0.1 | 22.0 ± 2.8 | **28.0 ± 4.2*** | **49.5 ± 2.1*** | 2.0 ± 0.0 |
| **24hrs** | | | | | |
| 0 | 1.9 ± 0.0 | 14.0 ± 1.4 | 14.5 ± 2.1 | 15.5 ± 0.7 | 0.0 ± 0.0 |
| 0.01 | 1.9 ± 0.0 | 16.0 ± 1.4 | 16.5 ± 2.1 | 16.5 ± 0.7 | 0.5 ± 0.7 |
| 0.025 | 1.8 ± 0.0 | 17.5 ± 0.7 | 17.5 ± 0.7 | 18.5 ± 0.7 | 0.0 ± 0.0 |
| 0.1 | 1.8 ± 0.0 | **24.5 ± 0.7*** | **19.0 ± 1.4*** | **53.0 ± 4.2*** | 2.5 ± 0.7 |
| **48hrs** | | | | | |
| 0 | 2.1 ± 0.1 | 13.5 ± 0.7 | 15.5 ± 0.7 | 17.0 ± 1.4 | 1.0 ± 1.4 |
| 0.01 | 1.7 ± 0.0 | 21.5 ± 5.0 | 24.5 ± 7.8 | 18.0 ± 2.8 | 0.5 ± 0.7 |
| 0.025 | 1.6 ± 0.0 | **58.5 ± 3.5*** | **63.5 ± 6.4*** | 15.0 ± 4.2 | 1.0 ± 1.4 |
| 0.1 | 1.1 ± 0.0 | - | - | - | - |

^1^50-60 000 cells were seeded in 6 well plates with 3 ml RPMI (4% FBS), subsequently different solutions of the test compound (dissolved in DMSO) were added. After treatment for different time periods, the cells was washed and processed as described in the Material and Method section. Numbers indicate means ± SD of values obtained in a representative experiment with two cultures per experimental point. For further details see table S1 A.

Table S1 C. Impact of different exposure periods on the results of cytokinesis block MN assays with H_2_O_2_ (4% FBS RPMI).^1^

| **Time/µM** | **Endpoint*** | | | | |
| --- | --- | --- | --- | --- | --- |
|  | **CBPI** | **BN-MN (‰)** | **MN(‰)** | **Buds (‰)** | **Bridges (‰)** |
| **4hrs** | | | | | |
| 0 | 1.8 ± 0.0 | 14.5 ± 2.1 | 14.5 ± 2.121 | 11.5 ± 3.5 | 0.0 ± 0.0 |
| 10 | 1.7 ± 0.1 | 17.0 ± 1.4 | 17.5 ± 0.707 | 10.0 ± 1.4 | 0.0 ± 0.0 |
| 25 | 1.6 ± 0.0 | **24.0 ± 1.4*** | **24.0 ± 1.4*** | 12.5 ± 2.1 | 0.0 ± 0.0 |
| 40 | 1.2 ± 0.1 | - | - | - | - |
| **24hrs** | | | | | |
| 0 | 1.8 ± 0.0 | 17.0 ± 1.4 | 19.5 ± 2.1 | 13.5 ± 0.7 | 0.5 ± 0.7 |
| 0.4 | 1.8 ± 0.0 | 23.0 ± 1.4 | 21.5 ± 0.7 | 19.0 ± 1.4 | 1.5 ± 0.7 |
| 2 | 1.7 ± 0.0 | 26.0 ± 4.2 | 25.0 ± 2.8 | **23.0 ± 1.4*** | 3.5 ± 0.7 |
| 10 | 1.7 ± 0.0 | **34.0 ± 4.2*** | **33.0 ± 2.8*** | **32.0 ± 2.8*** | 9.5 ± 0.7 |
| **48hrs** | | | | | |
| 0 | 1.9 ± 0.0 | 17.5 ± 2.1 | 18.5 ± 2.1 | 20.0 ± 1.4 | 0.0 ± 0.0 |
| 0.4 | 1.8 ± 0.0 | 18.0 ± 2.8 | 18.0 ± 2.8 | 16.0 ± 1.4 | 0.0 ± 0.0 |
| 2 | 1.8 ± 0.1 | 22.0 ± 2.8 | 23.5 ± 3.5 | 16.0 ± 2.8 | 0.0 ± 0.0 |
| 10 | 1.8 ± 0.0 | **28.0 ± 4.2*** | **31.5 ± 5.0*** | 25.0 ± 1.4 | 2.5 ± 0.7 |

^1^50-60 000 cells were seeded in 6 well plates with 3 ml RPMI (4% FBS), subsequently different solutions of the test compound (dissolved in medium) were added. After treatment for different time periods, the cells was washed and processed as described in the Material and Method section. Numbers indicate means ± SD of values obtained in a representative experiment with two cultures per experimental point. For further details see table S1 A.

Table S1 D. Impact of different exposure periods on the results of cytokinesis block MN assays with MMS (4% FBS RPMI).^1^

| **Time µg/ml** | **Endpoint*** | | | | |
| --- | --- | --- | --- | --- | --- |
|  | **CBPI** | **BN-MN (‰)** | **MN(‰)** | **Buds (‰)** | **Bridges (‰)** |
| 4hrs | | | | | |
| 0 | 1.9 ± 0.0 | 13.5 ± 0.7 | 14.5 ± 0.7 | 17.0 ± 1.4 | 0.0 ± 0.0 |
| 6 | 1.9 ± 0.1 | 20.5 ± 3.5 | 21.0 ± 4.2 | 20.5 ± 3.5 | 0.5 ± 0.7 |
| 10 | 1.7 ± 0.1 | **32.5 ± 3.5*** | **39.5 ± 0.7*** | **37.0 ± 4.2*** | 3.5 ± 0.7 |
| 40 | 1.5 ± 0.1 | **40.0 ± 7.1*** | **50.5 ± 7.8** | **42.0 ± 17.0*** | 3.5 ± 2.1 |
| **24hrs** | | | | | |
| 0 | 1.8 ± 0.0 | 17.0 ± 1.4 | 19.5 ± 2.1 | 13.5 ± 0.7 | 0.5 ± 0.7 |
| 0.4 | 1.7 ± 0.0 | 16.5 ± 0.7 | 16.5 ± 0.7 | 9.5 ± 0.7 | 0.5 ± 0.7 |
| 2 | 1.7 ± 0.0 | 21.5 ± 2.1 | 23.5 ± 2.1 | 17.5 ± 0.7 | 3.0 ± 0.0 |
| 10 | 1.6 ± 0.0 | **29.5 ± 2.1*** | **31.0 ± 2.8*** | **27.0 ± 1.4*** | 8.0 ± 1.4 |
| **48hrs** | | | | | |
| 0 | 1.9 ± 0.0 | 17.5 ± 2.1 | 18.5 ± 2.1 | 20.0 ± 1.4 | 0.0 ± 0.0 |
| 0.4 | 1.8 ± 0.0 | 18.0 ± 1.4 | 20.0 ± 1.4 | 19.0 ± 1.4 | 0.5 ± 0.7 |
| 2 | 1.9 ± 0.0 | 20.5 ± 0.7 | 23.5 ± 0.7 | 19.5 ± 0.7 | 2.5 ± 0.7 |
| 10 | 1.7 ± 0.0 | 24.0 ± 2.8 | 25.5 ± 3.5 | 27.0 ± 1.4 | 0.5 ± 0.7 |

^1^50-60 000 cells were seeded in 6 well plates with 3 ml RPMI (4% FBS), subsequently different solutions of the test compound (dissolved in medium) were added. After treatment for different time periods, the cells was washed and processed as described in the Material and Method section. Numbers indicate means ± SD of values obtained in a representative experiment with two cultures per experimental point. For further details see table S1 A.

Table S1 E. Impact of different exposure periods on the results of cytokinesis block MN assays with AFB1 (4% FBS RPMI).^1^

| **Time/µM** | **Endpoint*** | | | | | | | | | |
| --- | --- | --- | --- | --- | --- | --- | --- | --- | --- | --- |
|  | **CBPI** | **BN-MN (‰)** | | **MN(‰)** | | **Buds (‰)** | | **Bridges (‰)** | |  |
| **4hrs** | | | | | | | | | | |
| 0 | 1.7 ± 0.1 | | 19.0 ± 4.2 | | 20 ± 4.2 | | 16.5 ± 0.7 | | 0.5 ± 0.7 | |
| 5 | 1.6 ± 0.1 | | 24.6 ± 6.5 | | 24.5 ± 6.4 | | **29.5 ± 6.4*** | | 0.5 ± 0.7 | |
| 10 | 1.6 ± 0.0 | | 22.5 ± 6.4 | | 23.5 ± 6.4 | | **28.5 ± 6.4*** | | 2.5 ± 0.7 | |
| 20 | 1.6 ± 0.1 | | 25.0 ± 4.2 | | 27 ± 2.8 | | 25.5 ± 6.4 | | 1.5 ± 0.7 | |
| **24hrs** | | | | | | | | | | |
| 0 | 2.0 ± 0.1 | | 13.5 ± 1.4 | | 13.5 ± 1.4 | | 17.0 ± 2.8 | | 0.0 ± 0.0 | |
| 5 | 1.9 ± 0.1 | | 18.0 ± 1.4 | | 18.0 ± 1.4 | | 15.0 ± 1.4 | | 0.5 ± 0.7 | |
| 10 | 2.1 ± 0.1 | | **22.5 ± 2.1*** | | **22.5 ± 3.5*** | | 25.0 ± 1.4 | | 3.0 ± 1.4 | |
| 20 | 1.7 ± 0.0 | | **27.5 ± 4.2*** | | **29.0 ± 4.2*** | | **28.0 ± 1.4*** | | 2.0 ± 0.0 | |
| **48hrs** | | | | | | | | | | |
| 0 | 2.1 ± 0.1 | | 13.5 ± 0.7 | | 14.5 ± 0.7 | | 16.5 ± 0.7 | | 0.0 ± 0.0 | |
| 5 | 2.2 ± 0.1 | | 15.0 ± 1.4 | | 16.0 ± 2.8 | | 18.5 ± 2.1 | | 0.5 ± 0.7 | |
| 10 | 2.1 ± 0.1 | | **22.5 ± 3.5*** | | **25.0 ± 4.2*** | | **34.5 ± 7.8*** | | 3.5 ± 0.7 | |
| 20 | 1.6 ± 0.1 | | **28.0 ± 2.8*** | | **33.5 ± 2.1*** | | **52.5 ± 3.5*** | | 7.5 ± 0.7 | |

^1^50-60 000 cells were seeded in 6 well plates with 3 ml RPMI (4% FBS), subsequently different solutions of the test compound (dissolved in DMSO) were added. After treatment for different time periods, the cells was washed and processed as described in the Material and Method section. Numbers indicate means ± SD of values obtained in a representative experiment with two cultures per experimental point. For further details see table S1 A.

Table S1 F. Impact of different exposure periods on the results of cytokinesis block MN assays with B(a)P (4% FBS RPMI).^1^

| **Time/µM** | **Endpoint*** | | | | |
| --- | --- | --- | --- | --- | --- |
|  | **CBPI** | **BN-MN (‰)** | **MN(‰)** | **Buds (‰)** | **Bridges (‰)** |
| **4hrs** | | | | | |
| 0 | 1.7 ± 0.1 | 19.0 ± 4.2 | 20.0 ± 4.2 | 16.5 ± 0.7 | 0.5 ± 0.7 |
| 5 | 1.6 ± 0.1 | 17.5 ± 0.7 | 20.0 ± 1.4 | 23.5 ± 3.5 | 1.0 ± 1.4 |
| 10 | 1.6 ± 0.0 | 21.0 ± 4.2 | 24.0 ± 7.1 | **28.5 ± 3.5*** | 1.5 ± 0.7 |
| 20 | 1.6 ± 0.1 | **29.5 ± 3.5*** | **31.5 ± 6.4*** | **32.5 ± 3.5*** | 1.5 ± 2.1 |
| **24hrs** | | | | | |
| 0 | 1.9 ± 0.1 | 13.5 ± 0.7 | 14.5 ± 0.7 | 11.0 ± 1.4 | 0.0 ± 0.0 |
| 2.5 | 1.7 ± 0.1 | 16.5 ± 0.7 | 16.5 ± 0.7 | 12.5 ± 2.1 | 0.5 ± 0.7 |
| 5 | 1.4 ± 0.1 | **27.5 ± 2.1*** | **35.0 ± 1.4*** | **27.0 ± 1.4*** | 1.5 ± 0.7 |
| 10 | 1.2 ± 0.1 | - | - | - | - |
| **48hrs** | | | | | |
| 0 | 1.9 ± 0.0 | 10.5 ± 0.7 | 11.5 ± 0.7 | 11.0 ± 4.2 | 0.0 ± 0.0 |
| 0.4 | 1.9 ± 0.0 | 15.5 ± 0.7 | 16.0 ± 0.0 | 14.0 ± 1.4 | 0.0 ± 0.0 |
| 2.5 | 1.6 ± 0.1 | **27.5 ± 2.1*** | **35.0 ± 1.4*** | 17.5 ± 0.7 | 0.5 ± 0.7 |
| 5 | 1.1 ± 0.1 | - | - | - | - |

^1^50-60 000 cells were seeded in 6 well plates with 3 ml RPMI (4% FBS), subsequently different solutions of the test compound (dissolved in DMSO) were added. After treatment for different time periods, the cells was washed and processed as described in the Material and Method section. Numbers indicate mean ± SD of values obtained in a representative experiment with two cultures per experimental point. For further details see table S1 A.

Table S1 G. Impact of different exposure periods on the results of cytokinesis block MN assays with IQ (4% FBS RPMI).^1^

| **Time/µM** | **Endpoint*** | | | | |
| --- | --- | --- | --- | --- | --- |
|  | **CBPI** | **BN-MN (‰)** | **MN(‰)** | **Buds (‰)** | **Bridges (‰)** |
| **4hr** | | | | | |
| 0 | 1.9 ± 0.0 | 14.0 ± 1.4 | 14.5 ± 0.7 | 11.5 ± 3.5 | 0.0 ± 0.0 |
| 25 | 1.9 ± 0.1 | 16.0 ± 2.8 | 16.0 ± 2.8 | 17.5 ± 2.1 | 0.5 ± 0.7 |
| 50 | 1.8 ± 0.1 | 18.0 ± 4.2 | 19.0 ± 4.2 | 16.5 ± 0.7 | 0.5 ± 0.7 |
| 100 | 1.9 ± 0.0 | **23.5 ± 5.0*** | **24.5 ± 5.0*** | **21.5 ± 2.1*** | 1.5 ± 0.7 |
| **24hrs** | | | | | |
| 0 | 2.0 ± 0.1 | 16.0 ± 2.8 | 17.5 ± 3.5 | 7.5 ± 0.7 | 0.5 ± 0.7 |
| 25 | 1.9 ± 0.0 | 18.5 ± 0.7 | 19.5 ± 0.7 | 10.5 ± 2.1 | 0.0 ± 0.0 |
| 50 | 1.7 ± 0.0 | **26.0 ± 1.4*** | 26.5 ± 0.7 | **18.5 ± 2.1*** | 2.0 ± 0.0 |
| 100 | 2.0 ± 0.1 | **39.0 ± 5.7*** | **44.0 ± 1.4*** | **32.5 ± 5.0*** | 5.5 ± 0.7 |
| **48hrs** | | | | | |
| 0 | 1.9 ± 0.0 | 18.0 ± 1.4 | 19.0 ± 1.4 | 19.0 ± 1.4 | 0.5 ± 0.7 |
| 25 | 1.9 ± 0.1 | 20.5 ± 2.1 | 21.5 ± 2.1 | 22.0 ± 4.2 | 0.0 ± 0.0 |
| 50 | 1.8 ± 0.1 | **32.0 ± 5.7*** | **32.5 ± 6.4*** | **29.5 ± 2.1*** | 1.0 ± 0.0 |
| 100 | 1.7 ± 0.1 | **40.0 ± 2.8*** | **47.5 ± 3.5*** | **35.5 ± 3.5*** | 3.0 ± 1.4 |

^1^50-60 000 cells were seeded in 6 well plates with 3 ml RPMI (4% FBS), subsequently different solutions of the test compound (dissolved in DMSO) were added. After treatment for different time periods, the cells was washed and processed as described in the Material and Method section. Numbers indicate means ± SD of values obtained in a representative experiment with two cultures per experimental point. For further details see table S1 A.

Table S1 H. Impact of different exposure periods on the results of cytokinesis block MN assays with NDMA (4% FBS RPMI).^1^

| **Time/mM** | **Endpoint*** | | | | |
| --- | --- | --- | --- | --- | --- |
|  | **CBPI** | **BN-MN (‰)** | **MN(‰)** | **Buds (‰)** | **Bridges (‰)** |
| **4hrs** | | | | | |
| 0 | 1.9 ± 0.0 | 16.5 ± 2.1 | 18.0 ± 2.8 | 15.5 ± 7.8 | 0.5 ± 0.7 |
| 50 | 1.7 ± 0.1 | 21.0 ± 1.4 | 21.5 ± 0.7 | 16.5 ± 2.1 | 0.5 ± 0.7 |
| 100 | 1.7 ± 0.0 | 24.0 ± 2.8 | 26.5 ± 0.7 | 20.0 ± 1.4 | 0.5 ± 0.7 |
| 200 | 1.5 ± 0.0 | **26.5 ± 2.1*** | **27.5 ± 0.7*** | **25.5 ± 5.0*** | 0.5 ± 0.7 |
| **24hrs** | | | | | |
| 0 | 1.9 ± 0.1 | 19.0 ± 2.8 | 19.5 ± 2.1 | 4.0 ± 0.0 | 0.0 ± 0.0 |
| 25 | 1.8 ± 0.1 | 20.5 ± 0.7 | 20.5 ± 0.7 | 7.0 ± 1.4 | 0.0 ± 0.0 |
| 50 | 1.8 ± 0.1 | 21.5 ± 5.0 | 22.5 ± 3.5 | **9.5 ± 2.1*** | 0.5 ± 0.7 |
| 100 | 1.7 ± 0.0 | **30.0 ± 2.8*** | **32.0 ± 2.8*** | **13.0 ± 4.2*** | 3.5 ± 0.7 |
| **48hrs** | | | | | |
| 0 | 1.9 ± 0.0 | 18.0 ± 1.4 | 19.0 ± 1.4 | 19.0 ± 1.4 | 0.5 ± 0.7 |
| 25 | 1.8 ± 0.0 | 20.5 ± 0.7 | 21.5 ± 2.1 | 23.0 ± 2.8 | 0.5 ± 0.7 |
| 50 | 1.8 ± 0.0 | 22.0 ± 4.2 | 23.5 ± 5.0 | 27.0 ± 4.2 | 1.0 ± 1.4 |
| 100 | 1.7 ± 0.1 | **40.5 ± 2.1*** | **53.0 ± 4.2*** | **35.5 ± 3.5*** | 3.0 ± 1.4 |

^1^50-60 000 cells were seeded in 6 well plates with 3 ml RPMI (4% FBS), subsequently different solutions of the test compound (dissolved in medium) were added. After treatment for different time periods, the cells was washed and processed as described in the Material and Method section. Numbers indicate means ± SD of values obtained in a representative experiment with two cultures per experimental point. For further details see table S1 A.

Table S1 I. Impact of different exposure periods on the results of cytokinesis block MN assays with PhiP (4% FBS RPMI).^1^

| **Time/µM** | **Endpoint*** | | | | |
| --- | --- | --- | --- | --- | --- |
|  | **CBPI** | **BN-MN (‰)** | **MN (‰)** | **Buds (‰)** | **Bridges (‰)** |
| **4hrs** | | | | | |
| 0 | 1.7 ± 0.1 | 19.0 ± 4.2 | 20.0 ± 4.2 | 16.5 ± 0.7 | 0.5 ± 0.7 |
| 25 | 1.7 ± 0.1 | 23.5 ± 0.7 | 23.5 ± 0.7 | 18.0 ± 5.7 | 0.5 ± 0.7 |
| 50 | 1.6 ± 0.2 | 22.5 ± 2.1 | 22.5 ± 2.1 | 19.0 ± 7.1 | 2.0 ± 1.4 |
| 100 | 1.7 ± 0.0 | 26 ± 1.4 | 28 ± 1.4 | 23.5 ± 6.4 | 1.5 ± 0.7 |
| **24hrs** | | | | | |
| 0 | 2.0 ± 0.1 | 17.5 ± 3.5 | 17.5 ± 3.5 | 7.5 ± 0.7 | 0.5 ± 0.7 |
| 25 | 1.9 ± 0.1 | 17.5 ± 2.1 | 17.5 ± 2.1 | 10.5 ± 0.7 | 0.0 ± 0.0 |
| 50 | 1.8 ± 0.0 | **26.5 ± 0.7*** | **30.0 ± 1.4*** | **24.5 ± 0.7*** | 1.5 ± 0.7 |
| 100 | 1.9 ± 0.0 | **49.5 ± 2.1*** | **49.5 ± 2.1*** | **34.0 ± 1.4*** | 3.0 ± 0.0 |
| **48hrs** | | | | | |
| 0 | 1.9 ± 0.0 | 18.0 ± 1.4 | 19.0 ± 1.4 | 19.0 ± 1.4 | 0.5 ± 0.7 |
| 25 | 1.9 ± 0.1 | 20.0 ± 1.4 | 20.5 ± 0.7 | 20.5 ± 3.5 | 0.0 ± 0.0 |
| 50 | 1.9 ± 0.0 | 20.5 ± 3.5 | 21.5 ± 3.5 | 22.0 ± 4.2 | 0.5 ± 0.7 |
| 100 | 1.8 ± 0.2 | 22.0 ± 1.4 | 24.0 ± 1.4 | **29.5 ± 0.7*** | 0.5 ± 0.7 |

^1^50-60 000 cells were seeded in 6 well plates with 3 ml RPMI (4% FBS), subsequently different solutions of the test compound (dissolved in DMSO) were added. After treatment for different time periods, the cells was washed and processed as described in the Material and Method section. Numbers indicate means ± SD of values obtained in a representative experiment with two cultures per experimental point. For further details see table S1 A.
