# Supplementary material for: Cytome micronucleus assays with a metabolically competent human derived liver cell line (Huh6): A promising approach for routine testing of chemicals?
Source: Environ Mol Mutagen. 2018 Nov 8;60(2):134–44. doi: 10.1002/em.22254 (PMC6492180; doi:10.1002/em.22254)
Supplement: Supplementary file 3 — Table S2. Results of cytome MN assays with different diagnostic mutagens (10% FBS RPMI)1 [file EM-60-134-s003.docx]

Table S2 Results of cytome MN assays with different diagnostic mutagens (10% FBS RPMI)^1^

| **Test compound** | **Dose** |  | | **Endpoint*** | | | |
| --- | --- | --- | --- | --- | --- | --- | --- |
|  |  | **CBPI** | **BN-MN** | | **MN** | **Buds** | **Bridges** |
| **CDDP (µg/ml)** | 0 | 2.0 ± 0.0 | 17.0 ± 1.4 | | 17.0 ± 1.4 | 15.0 ± 1.4 | 0.0 ± 0.0 |
|  | 0.5 | 1.9 ± 0.0 | **38.0 ± 1.4*** | | **40.5 ± 0.7*** | **32.0 ± 5.7*** | 0.5 ± 0.7 |
|  | 1 | 1.9 ± 0.1 | **46.5 ± 2.1*** | | **50.0 ± 1.4*** | **49.0 ± 2.8*** | - 1. ± 2.1 |
| **Etoposide (µg/ml)** | 0 | 2.2 ± 0.1 | 20.5 ± 3.5 | | 21.5 ± 3.5 | 38.0 ± 4.2 | 0.5 ± 0.7 |
|  | 0.1 | 1.9 ± 0.0 | **57.5 ± 3.5*** | | **61.0 ± 4.2*** | **80.0 ± 2.8*** | 6.5 ± 0.7 |
| **H_2_O_2_ (µM)** | 0 | 2.0 ± 0.0 | 17.0 ± 1.4 | | 17.0 ± 1.4 | 15.0 ± 1.4 | 0.0 ± 0.0 |
|  | 10 | 1.9 ± 0.0 | **32.5 ± 3.5*** | | **33.5 ± 3.5*** | **40.0 ± 2.8*** | 4.5 ± 0.7 |
| **MMS (µg/ml)** | 0 | 1.9 ± 0.0 | 15.0 ± 1.4 | | 16.0 ± 2.8 | 15.0 ± 1.4 | 0.0 ± 0.0 |
|  | 10 | 1.8 ± 0.0 | **27.5 ± 2.1*** | | **30.0 ± 1.4*** | 20.0 ± 2.8 | 0.0 ± 0.0 |
| **AFB1 (µM)** | 0 | 2.2 ± 0.1 | 20.5 ± 3.5 | | 21.5 ± 3.5 | 38.0 ± 4.2 | 1.5 ± 0.7 |
|  | 10 | 2.0 ± 0.1 | **57.0 ± 5.7*** | | **60.5 ± 3.5*** | **61.5 ± 5.0*** | 7.0 ± 1.4 |
|  | 20 | 1.8 ± 0.1 | **66.5 ± 2.1*** | | **69.0 ± 1.4*** | **69.5 ± 6.4*** | 7.5 ± 0.7 |
| **B(a)P (µM)** | 0 | 2.0 ± 0.1 | 14.5 ± 0.7 | | 14.5 ± 0.7 | 10.0 ± 2.8 | 0.5 ± 0.7 |
|  | 5 | 1.7 ± 0.0 | **51 ± 4.2*** | | **54.5 ± 5.0*** | 13.5 ± 6.4 | 0.5 ± 0.7 |
|  | 10 | 1.3 ± 0.1 | - | | - | - | - |
| IQ **(µM)** | 0 | 2.2 ± 0.1 | 20.5 ± 3.5 | | 21.5 ± 3.5 | 38 ± 4.2 | 0.5 ± 0.7 |
|  | 50 | 1.9 ± 0.0 | **47.5 ± 3.5*** | | **51.0 ± 1.4*** | 42 ± 2.8 | 0 ± 0.0 |
|  | 100 | 1.8 ± 0.1 | **63.5 ± 6.4*** | | **68.5 ± 5.0*** | 49 ± 1.4 | 0.5 ± 0.7 |
| **NDMA (µM)** | 0 | 2.2 ± 0.1 | 20.5 ± 3.5 | | 21.5 ± 3.5 | 38.0 ± 4.2 | 0.5 ± 0.7 |
|  | 100 | 1.9 ± 0.0 | **33.0 ± 2.8*** | | **34.5 ± 5.0*** | 28.0 ± 2.8 | 3.5 ± 0.7 |
| **PhiP (µM)** | 0 | 2.2 ± 0.1 | 20.5 ± 3.5 | | 21.5 ± 3.5 | 38.0 ± 4.2 | 0.5 ± 0.7 |
|  | 50 | 2.0 ± 0.1 | **50.0 ± 2.8*** | | **56.5 ± 2.1*** | 50.0 ± 1.4 | 1.0 ± 1.4 |
|  | 100 | 1.8 ± 0.0 | **55.0 ± 11.3*** | | **61 ± 12.7*** | **60.0 ± 7.1*** | 1.5 ± 0.7 |

^1^The experiments were conducted essentially as described in the footnote of Table S1A-I. *p≤0.05
